# Albumin binding ligands and albumin conjugate uptake by cancer cells

**DOI:** 10.1186/1758-5996-3-11

**Published:** 2011-06-15

**Authors:** Eva Frei

**Affiliations:** 1 Division of Preventive Oncology, German Cancer Research Center and National Center for Tumor Diseases, Im Neuenheimer Feld 280, 69120 Heidelberg, Germany

**Keywords:** Insulin detemir, aminofluorescein-albumin, methotrexate-albumin, clathrin-heavy-chain, lysosomes, fatty acids

## Abstract

The scope of this short review is to summarise the knowledge gleaned from the fate of drugs transported by albumin upon contact with the target cancer cell or cells in inflamed tissues. The authors expertise covers covalently bound drugs and their cellular uptake and release from albumin. This review therefore aims to deduce what will happen to drugs such as insulin detemir which is considered to bind non-covalently to albumin and may have a fate similar to fatty acids transported by albumin.

## Introduction

**Figure 1:**
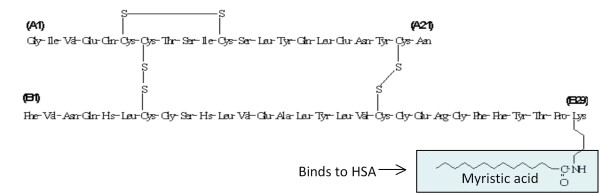
**Insulin detemir**. Thr at position 30 in the B chain in human insulin was cleaved off and myristic acid covalently attached to Lys 29 in the B chain to enable albumin (HSA) binding.

Insulin detemir was developed as a long acting drug to achieve a continuous and steady blood level of insulin rather than the peaks achieved with postprandial injections of conventional insulin. The molecule was modified by eliminating Thr 30 and adding a C14 fatty acid (myristic acid) by a chemically very stable amide bond to the epsilon amine of Lys 29 on the B chain of human insulin [Figure 1]. The reason for the long duration of action of insulin detemir of about 20 hrs compared to 12- 16 hrs for Neutral Protamine Hagedorn (NPH) insulin is considered to be its binding to human serum albumin (HSA) to be released either at the insulin receptor or in circulation [1]. The mechanisms of its binding and even more so of its release are, however, not very clear and the author of this short review will try to critically evaluate the literature available on the subject in view of the properties of albumin loaded with either fatty acids or covalently bound drugs and its cellular uptake.

### Albumin as drug carrier

Albumin is the major protein in blood plasma, its blood levels are tightly regulated because of its importance for homeostasis [2]. One of its functions is the transport of physiological non or poorly water soluble molecules like fatty acids, steroids and drugs. These are bound to distinct hydrophobic pockets in the albumin molecule, which has a very rigid and stable three dimensional structure due to 17 S-S bonds. In addition albumin contains very few aromatic amino acids but many amino acids with functional groups like amino and carboxyl groups, these contribute to stabilising the blood pH. In addition to greatly increasing its stability against solvents, denaturing agents and heat - an unusual property for a protein of 66 kDa molecular weight - these functional groups make albumin amenable to chemically link drugs. The body is, however, very sensitive to the structure of albumin and rapidly eliminates any molecules which have not retained the native structure of albumin [3]. Therefore only albumin-drug carriers with a molar loading ratio of on average 1.4 moles of drug per mole of albumin are physiologically stable and attain half-lives similar to native albumin [4].

### Cellular uptake of albumin

Under conditions of cellular stress, as in growing tumour tissue, albumin is taken up by cells as a source of amino acids and energy [5]. This was shown with albumin covalently labelled with a radiolabel or a fluorescent dye, such as aminofluorescein, at a molar ratio of approximately one. The fate of the protein can thus be traced in cellular systems, but also *in vivo *in experimental animals and, with the fluorescent conjugate, also in patients.

Such experiments showed that albumin is taken up by endocytosis into the lysosomal compartment of cells, where it is degraded. The exact mechanism leading to endocytosis, i.e. which membrane receptor is responsible and if the process is indeed receptor mediated is not yet entirely elucidated. In endothelia a gp60 protein, albondin, binds albumin and is a shuttle enabling transcytosis of albumin into the underlying tissue [6]. As of now there is no evidence that this protein is expressed on tumour cells and it is not very likely that it is the receptor leading to lysosomal targeting of albumin. The protein SPARC (secreted protein, acidic and rich in cysteine) also binds native albumin and antibodies directed against gp60 [6]. SPARC has therefore been suggested to be involved in cellular albumin uptake [7]. SPARC is a protein found expressed at higher levels in tumour tissue than in healthy organs [7]. There is no direct experimental proof of SPARC-mediated cellular albumin uptake. Our own data have shown albumin binding proteins to be expressed on various human tumour cell lines derived from solid tumours and on leukemic cells [8]. Whether these proteins indeed mediate albumin uptake has not been shown. Clathrin heavy chain si-RNA used to inhibit clathrin-mediated endocytosis did not change the uptake of labelled albumin into tumour cells, but inhibited the uptake of transferrin which was used as positive control. Hypertonic sucrose in the media very effectively inhibited uptake of both, labelled albumin and transferrin, but also of dextran (V. Pohl personal communication Ph.D. thesis). Together these data point to a possible fluid-phase uptake of albumin, because hypertonic conditions inhibit both, clathrin and fluid-phase endocytosis; for the latter dextran is a marker [9]. Since albumin is such a ubiquitous molecule any experimental setting to show the mechanism of its uptake pathway is very difficult to perform.

In experimental animals bearing solid tumours radioactively-or fluorescently labelled albumin was taken up into tumours specifically [3]. The same specific uptake of albumin into inflamed joints of mice in the collagen induced arthritis model as into tumour tissue was seen [10], emphasizing the similarity of physiological processes in inflammation and tumour growth, such as an increased catabolism of proteins [5].

### Clinical studies

In a clinical study testing aminofluorescein-labelled albumin as a diagnostic agent for fluorescence guided brain surgery, we could show that all glioblastoma patients included in the study accumulated fluorescence and thereby albumin into the tumour and not in the normal brain, the oedema or necrosis. The fluorescence of the tumour facilitated brain surgery [11].

The pharmacokinetics performed on these patients showed a terminal plasma half-life of 12.8 days [12], which is similar to the half-life reported for native albumin of 19 days [2], emphasizing the importance of a careful loading procedure of albumin with drug or tag. Higher loading ratios of drug to albumin had been shown to lead to accumulation in the liver, rather than in the tumour. Such modified albumins are rapidly eliminated by the reticulo-endothelial system of the liver [3].

Clinical studies with methotrexate covalently linked by an amide bond to a lysine in human serum albumin showed moderate efficiency against solid tumours in phase I and phase II clinical studies [13,14]. Solid tumours such as renal cell carcinoma, which are usually not treated with methotrexate, were sensitive to this drug linked to albumin. Also here, a long plasma half-life of the drug protein conjugate was found, probably the reason for the observed effect together with the specific enrichment in tumour tissue. In *in vitro *experiments the amide bond was found to be extremely stable against enzymatic degradation resulting in a methotrexate linked to lysine as the final metabolite after proteolysis [15].

### Insulin detemir and albumin binding

Also in this molecule an amide bond links the fatty acid myristate to a lysine at position 29 on the B chain of insulin resulting in a longer half-life of insulin detemir than insulin in blood. The reason for this is postulated to be binding of the fatty acid to albumin, because of fatty acid binding sites on albumin. These are discrete regions on the molecule accommodating long chain fatty acids, which are essential and have chain lengths between C16 and C20 either saturated or with up to 4 double bonds. Medium chain fatty acids (C6-C14) are normally not transported by albumin, because they are much more water soluble and are normally not found outside cells [2]. Binding affinities of such medium chain fatty acids are much lower than of the long chain fatty acids for which 2 strong and 4 weaker binding sites on albumin have been found. Medium chain fatty acids are excluded from these primary strong binding sites. Initial binding is a rapid ionic interaction of the carboxyl group with albumin leading to a hydrophobic interaction of the fatty acid chain with the loops of the protein. Non charged ligands bind less tightly, this would also be true for insulin-detemir, because the carboxyl group of its fatty acid is masked in an amide bond, and therefore no extra charge compared to insulin is added. Insulin itself binds only weakly to albumin.

The glucose lowering potency of insulin detemir is much lower than of human insulin, the reason being that binding of the former to the insulin receptor is much weaker. This has been shown recently by Sorensen who compared the displacement of radioactively labelled insulin from the receptor by insulin and insulin detemir [16]. The authors also show that this low binding affinity for the insulin receptor can be further decreased by adding fatty acid free albumin to the incubation. They needed approximately four fold higher insulin detemir concentrations for half maximal displacement of radioactive insulin from the insulin receptor, but not of insulin, if albumin was increased from 0.1% to 1% in the cell medium at insulin detemir concentrations around 10^-7 ^M. It is, however, not clear if indeed albumin binding of insulin detemir is the reason for this observation, because a 0.1% albumin solution is 15 μM and at 1% 150 μM, i.e. 150 or 1500 times more concentrated than the insulin detemir added. In addition the albumin was essentially fat-free, therefore expected to bind at least 3 moles of ligand per mole albumin [2].

The albumin binding studies performed by the same group showed displacement of insulin detemir by fatty acids with chain lengths of C12 and C16. At an insulin detemir to albumin ratio of one, 50% is displaced. Insulin detemir is therefore considered to bind to the medium to long fatty acid binding site [17]. The balance between albumin binding of insulin detemir and its displacement by long chain fatty acids in blood is difficult to estimate from these *in vitro *assays, since here fatty acid free albumin was used for a clear experimental set-up, but this species does not occur *in vivo*. The further fate of insulin detemir bound to albumin and the question whether it might be internalised is an educated guess. Fatty acids are „liberated" from albumin by various fatty acid transporters or fatty acid binding proteins, which however do not transport fatty acid esters or triglycerides [18]. Insulin detemir therefore is probably not a substrate for these proteins. If insulin detemir were bound covalently or more tightly than fatty acid, which is not the case, uptake of the albumin bound drug into lysosomes might occur. Insulin would then not be active anymore because proteases would degrade the carrier and the ligand. This might happen to a small extent and might contribute to the lower potency of insulin detemir compared to insulin. How likely such a cellular uptake is, however, difficult to deduce from the data available.

## Competing interests

The author declares that she has no competing interests.

## References

[B1] Hartman I Insulin analogs: impact on treatment success, satisfaction, quality of life, and adherence Clin Med Res 2008 6 54 67 10.3121/cmr.2008.793 18801953 PMC2572551

[B2] Peters T All about Albumin: Biochemistry, Genetics, and Medical Applications 1996 San Diego: Academic Press

[B3] Stehle G Sinn H Wunder A Schrenk HH Schutt S Maier-Borst W Heene DL The loading rate determines tumor targeting properties of methotrexate-albumin conjugates in rats Anticancer Drugs 1997 8 677 685 9311444

[B4] Neumann E Frei E Funk D Becker MD Schrenk HH Muller-Ladner U Fiehn C Native albumin for targeted drug delivery Expert Opin Drug Deliv 2010 7 915 925 10.1517/17425247.2010.498474 20586704

[B5] Stehle G Sinn H Wunder A Schrenk HH Stewart JC Hartung G Maier-Borst W Heene DL Plasma protein (albumin) catabolism by the tumor itself--implications for tumor metabolism and the genesis of cachexia Crit Rev Oncol Hematol 1997 26 77 100 10.1016/S1040-8428(97)00015-2 9298326

[B6] Schnitzer JE Oh P Albondin-mediated capillary permeability to albumin. Differential role of receptors in endothelial transcytosis and endocytosis of native and modified albumins J Biol Chem 1994 269 6072 6082 8119952

[B7] Desai NP Trieu V Hwang LY Wu R Soon-Shiong P Gradishar WJ Improved effectiveness of nanoparticle albumin-bound (nab) paclitaxel versus polysorbate-based docetaxel in multiple xenografts as a function of HER2 and SPARC status Anticancer Drugs 2008 19 899 909 10.1097/CAD.0b013e32830f9046 18766004

[B8] Fritzsche T Schnolzer M Fiedler S Weigand M Wiessler M Frei E Isolation and identification of heterogeneous nuclear ribonucleoproteins (hnRNP) from purified plasma membranes of human tumour cell lines as albumin-binding proteins Biochem Pharmacol 2004 67 655 665 10.1016/j.bcp.2003.09.027 14757165

[B9] Wang S Singh RD Godin L Pagano RE Hubmayr RD Endocytic response of type I alveolar epithelial cells to hypertonic stress Am J Physiol Lung Cell Mol Physiol 2011 300 L560 L568 10.1152/ajplung.00309.2010 21257731 PMC3075106

[B10] Wunder A Muller-Ladner U Stelzer EH Funk J Neumann E Stehle G Pap T Sinn H Gay S Fiehn C Albumin-based drug delivery as novel therapeutic approach for rheumatoid arthritis J Immunol 2003 170 4793 4801 12707361 10.4049/jimmunol.170.9.4793

[B11] Kremer P Fardanesh M Ding R Pritsch M Zoubaa S Frei E Intraoperative fluorescence staining of malignant brain tumors using 5-aminofluorescein-labeled albumin Neurosurgery 2009 64 53 60 19240573 10.1227/01.NEU.0000335787.17029.67

[B12] Ding R Frei E Fardanesh M Schrenk HH Kremer P Haefeli WE Pharmacokinetics of 5-Aminofluorescein-Albumin, a Novel Fluorescence Marker of Brain Tumors During Surgery J Clin Pharmacol 2011 51 672 678 10.1177/0091270010372626 20978277

[B13] Hartung G Stehle G Sinn H Wunder A Schrenk HH Heeger S Kranzle M Edler L Frei E Fiebig HH Heene DL Maier-Borst W Queisser W Phase I trial of methotrexate-albumin in a weekly intravenous bolus regimen in cancer patients. Phase I Study Group of the Association for Medical Oncology of the German Cancer Society Clin Cancer Res 1999 5 753 759 10213209

[B14] Vis AN van der Gaast A van Rhijn BW Catsburg TK Schmidt C Mickisch GH A phase II trial of methotrexate-human serum albumin (MTX-HSA) in patients with metastatic renal cell carcinoma who progressed under immunotherapy Cancer Chemother Pharmacol 2002 49 342 345 10.1007/s00280-001-0417-z 11914915

[B15] Wosikowski K Biedermann E Rattel B Breiter N Jank P Loser R Jansen G Peters GJ In vitro and in vivo antitumor activity of methotrexate conjugated to human serum albumin in human cancer cells Clin Cancer Res 2003 9 1917 1926 12738750

[B16] Sorensen AR Stidsen CE Ribel U Nishimura E Sturis J Jonassen I Bouman SD Kurtzhals P Brand CL Insulin detemir is a fully efficacious, low affinity agonist at the insulin receptor Diabetes Obes Metab 2010 12 665 673 10.1111/j.1463-1326.2010.01206.x 20590743

[B17] Kurtzhals P Havelund S Jonassen I Markussen J Effect of fatty acids and selected drugs on the albumin binding of a long-acting, acylated insulin analogue J Pharm Sci 1997 86 1365 1368 10.1021/js9701768 9423147

[B18] Abumrad N Coburn C Ibrahimi A Membrane proteins implicated in long-chain fatty acid uptake by mammalian cells: CD36, FATP and FABPm Biochim Biophys Acta 1999 1441 4 13 10526223 10.1016/s1388-1981(99)00137-7

